# Supplementary material for: Matrix directs trophoblast differentiation in a bioprinted organoid model of early placental development
Source: Nat Commun. 2025 Sep 12;16:8267. doi: 10.1038/s41467-025-62996-0 (PMC12432263; doi:10.1038/s41467-025-62996-0)
Supplement: Supplementary file 1 — Supplementary Information [file 41467_2025_62996_MOESM1_ESM.pdf]

Supplementary Information

Matrix directs trophoblast differentiation in a bioprinted organoid model of early placental development

Claire Richards[1,2], Hao Chen[3], Matthew O'Rourke[3], Ashley Bannister[1], Grace Owen[1], Alexander Volkerling[4], Arnab Ghosh[5,6], Catherine Gorrie[1], David Gallego-Ortega[7,8,9], Amy Bottomley[10], Matthew P Padula[1], Kristine McGrath1, Louise Cole[10], Philip Hansbro[3], Lana McClements[1, 2,#]

1. School of Life Sciences, Faculty of Science, University of Technology Sydney, NSW, Australia
2. Institute for Biomedical Materials and Devices, Faculty of Science, University of Technology Sydney, NSW, Australia
3. Centre for Inflammation, Centenary Institute and University of Technology Sydney, Faculty of Science, School of Life Sciences, Sydney, NSW, Australia
4. Inventia Life Science Pty Ltd, Sydney, NSW, Australia
5. School of Biomedical Sciences and Pharmacy, University of Newcastle, Callaghan, NSW, Australia
6. Centre for Drug Repurposing and Medicines Research Program, Hunter Medical Research Institute, New Lambton Heights, NSW, Australia
7. School of Biomedical Engineering, Faculty of Engineering and Information Technology, University of Technology Sydney, NSW, Australia
8. Garvan Institute of Medical Research, The Kinghorn Cancer Centre, Darlinghurst, NSW 2010, Australia
9. School of Clinical Medicine, Faculty of Medicine, University of New South Wales Sydney, Kensington, NSW 2052, Australia
10. Microbial Imaging Facility at the Australian Institute for Microbial Imaging, Faculty of Science, University of Technology Sydney, NSW, Australia

# Corresponding author: lana.mcclements@uts.edu.au

The PDF file contains:

Supplementary Figures 1-4.
Supplementary Tables 1-2.

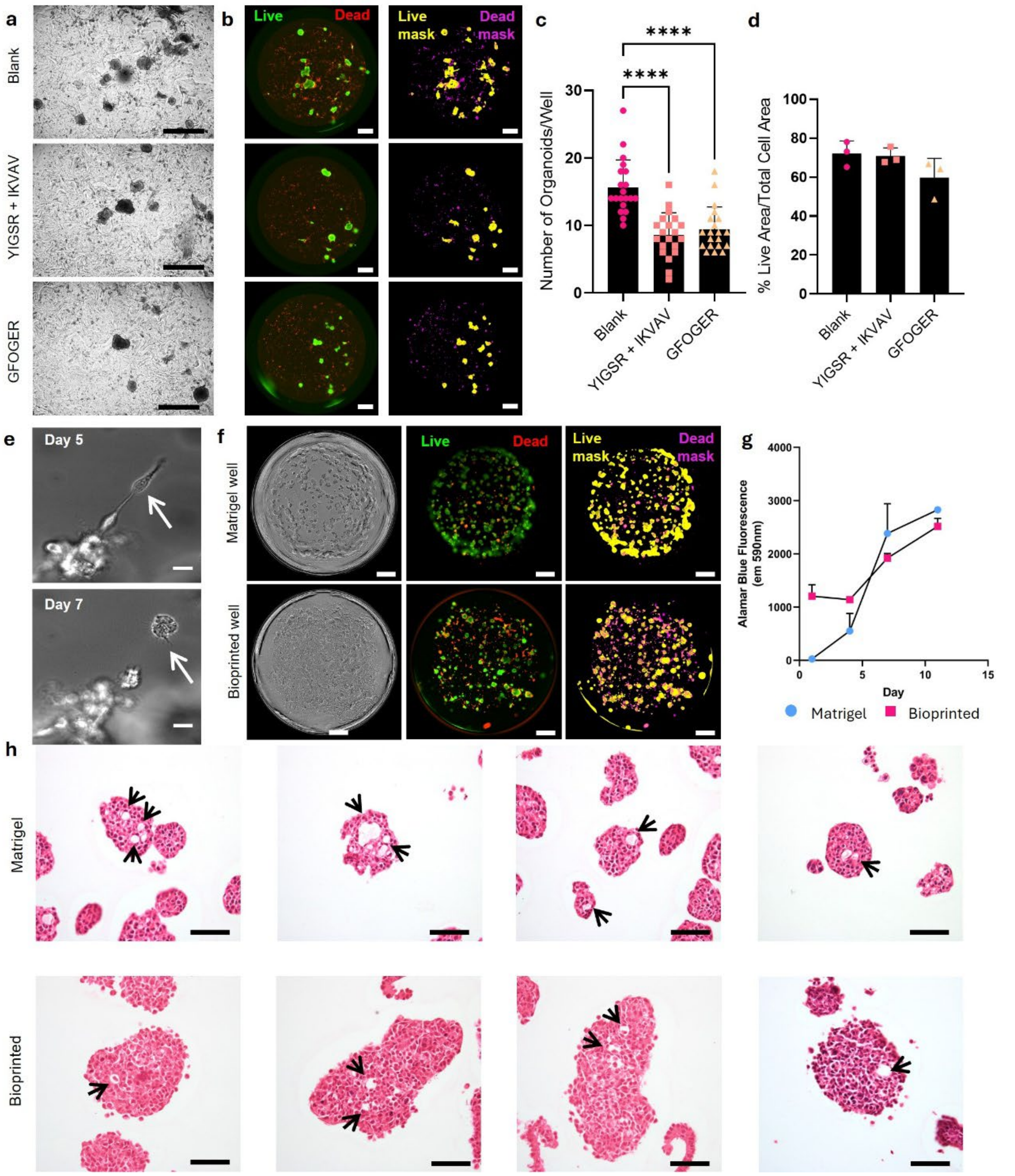

Supplementary Figure 1. Matrix selection for bioprinted hydrogel selection and further characterisation of Matrigel and bioprinted organoids. a, Cell viability images comparing organoids bioprinted in blank PEG matrix (no peptides added) or with YIGSR and IKVAV (α and β laminin chains) or GFOGER (collagen I) added. IncuCyte; scale bar = 900 μm. **b,** Live (green; yellow mask) and dead (red; magenta mask) cells in matrix; scale bar = 800 μm. **c,** Number of organoids counted per bioprint unit. One-way ANOVA with Kruskal Wallis, each data point represents a single bioprinted unit, ****p<0.0001. **d,** Viability determined by comparing area of live cells to all cells. One-way ANOVA with Kruskal Wallis, each data point represents a bioprint unit. **e,** Images of single cells (arrows) migrating out of an organoid over time; scale

bar = 10 μm. **f,** Whole well images of phase contrast, green or red fluorescence channels with analysis masks applied to calculate live and dead areas; scale bar = 800 μm. **g,** Total Alamar Blue fluorescence intensity measured at days 1, 4, 7 and 11 across three bioprint units. Data presented as mean ± SEM. **h,** Haematoxylin and eosin stained organoid sections from Matrigel or bioprinted conditions with cavities (black arrows); scale bar = 100 μm.

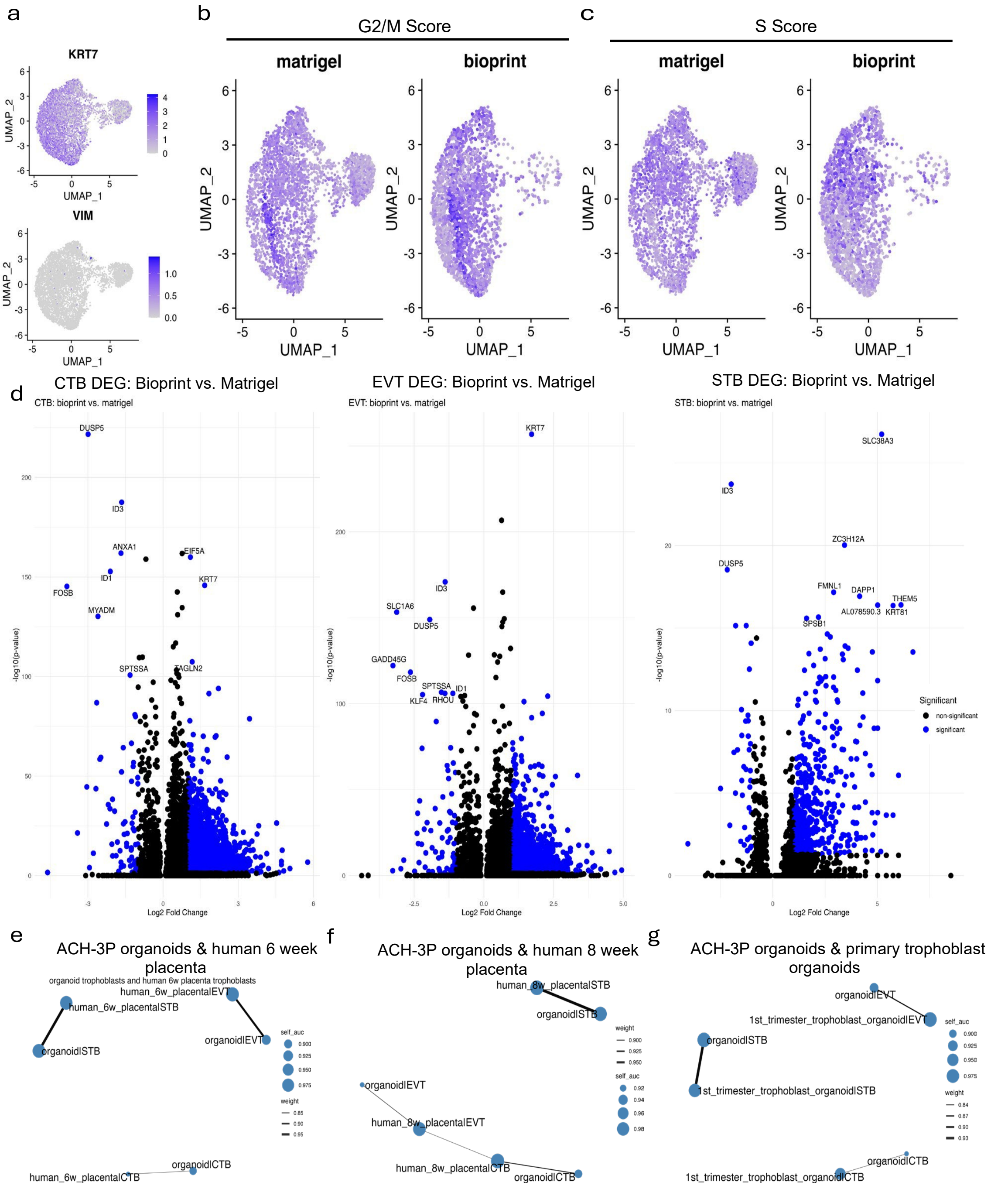

Supplementary Figure 2. Transcriptomic profiling of key identity and cell cycle markers in trophoblast organoids. Uniform manifold approximation and projection (UMAP) plots of **a,** cytokeratin 7 (KRT7) and vimentin (VIM). **b,** markers of G2/M and **c,** S phase in single cells from Matrigel and bioprinted organoids. **d,** Volcano plots displaying differentially expressed genes (DEGs) between CTB, EVT and STB subtypes of organoids. Genes more highly expressed in Matrigel organoids extend from the centre point to the left of the x-axis. Genes more highly expressed in bioprinted organoids extend from the centre point to the right of the x-axis. Significantly different

genes are denoted by a blue dot. Analysis calculated with FindMarkers with min.pct=0.25 and logfc.threshold=0.4 using the Wilcoxon algorithm. Annotations correspond to the 10 most significantly different genes in each comparison. **e-g,** Area under the receiver-operating-characteristic curve (AUROC) similarity networks between cell types from combined organoid transcriptomic dataset compared to published data. Priori threshold AUROC > 0.88.

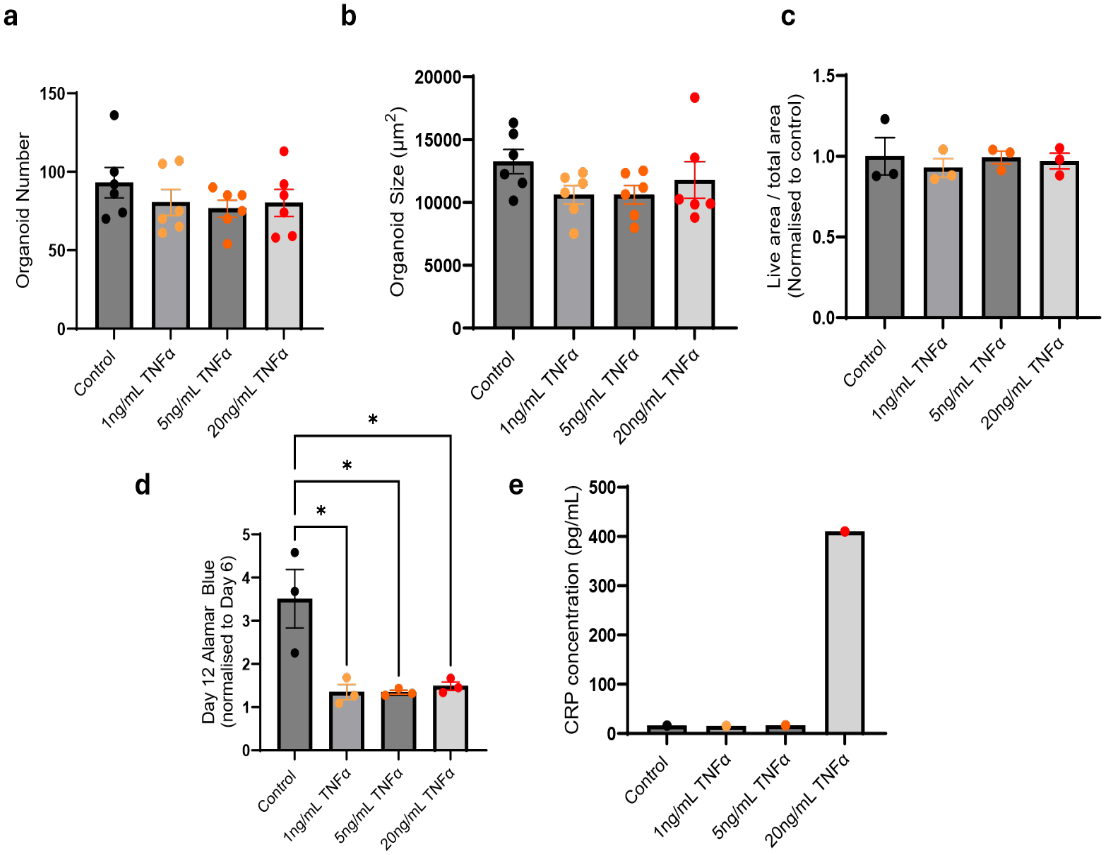

Supplementary Figure 3. Tumour necrosis factor-α (TNFα) concentration screen. a, Number of bioprint organoids after stimulation with varying concentrations of TNFα (n=6 bioprint units). **b,** Mean size of bioprinted organoids stimulated with varying concentrations of TNFα (n=6 bioprint units). **c,** Viability of bioprinted organoids stimulation with varying concentrations of TNFα (n=3 bioprint units). **d,** Relative Alamar Blue fluorescence normalised to Day 6 for respective conditions (n=3 bioprint units); *$p<0.05$ (Control vs 1ng/ml TNF-α, p=0.011; Control vs 5ng/ml TNF-α, p=0.0109; Control vs 20ng/ml TNF-α, p=0.0159). **e,** Concentration of C reactive protein (CRP) in conditioned medium pooled from bioprinted organoids stimulated with varying concentrations of TNFα. Data collected at Day 12 and plotted as mean ± SEM, points represent individual bioprint units (except e), one way ANOVA with Tukey's multiple comparison test.

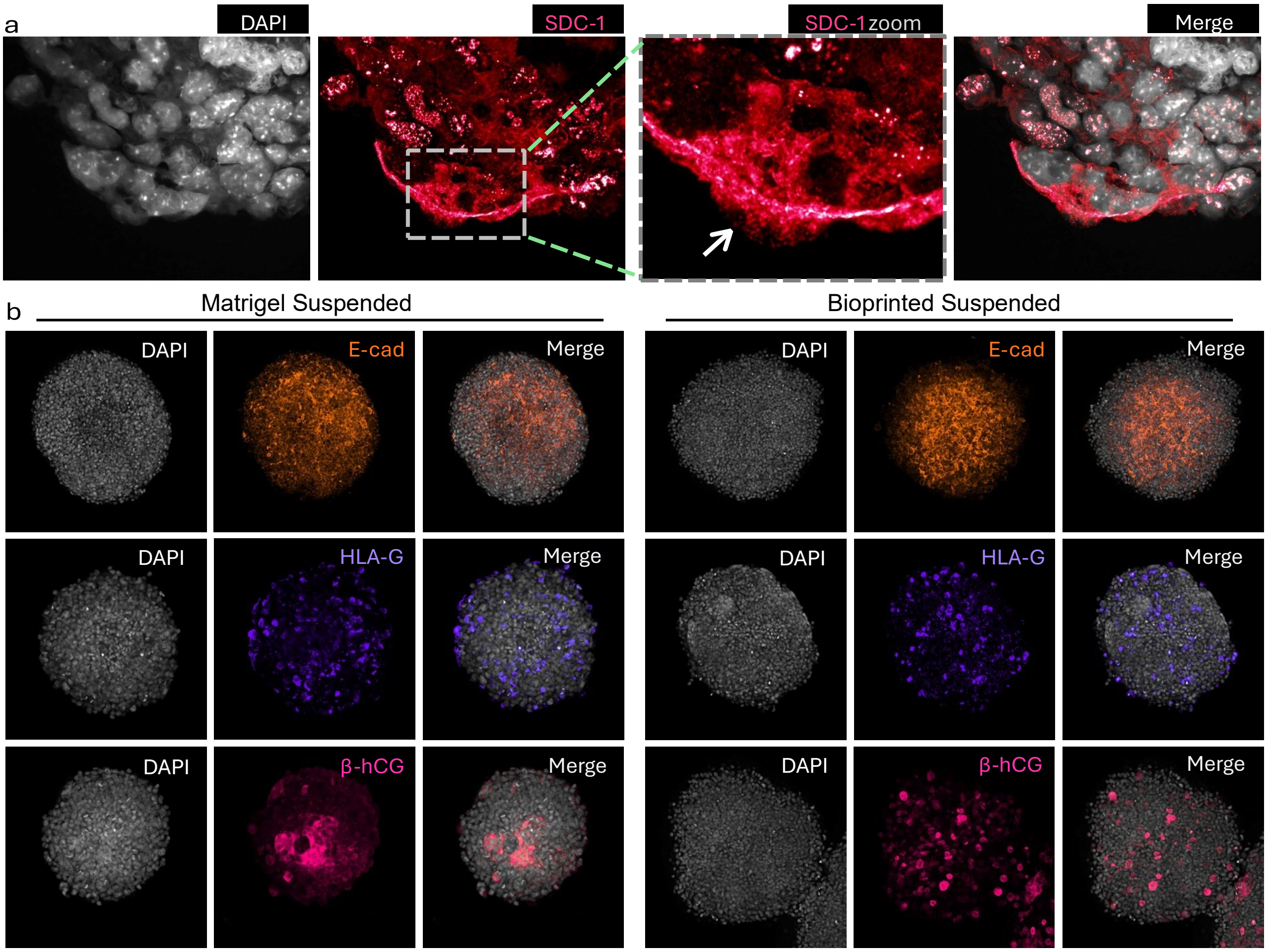

Supplementary Figure 4. Suspended organoids from Matrigel and bioprinted matrix conditions retain expression of major trophoblast subtype markers. a, Representative confocal image of a syncytial border on the periphery of a suspended Matrigel organoid labelled for DAPI (grey) and syndecan-1 (SDC-1, pop cherry). Microvillus-like structures are denoted by a white arrow; scale bar = 20 μm. **b,** Confocal MIPs of suspended organoids immunolabelled for DAPI (grey), E-cadherin (E-cad, orange), human leukocyte antigen G (HLA-G, purple) or beta human chorionic gonadotropin (β-hCG, pink); scale bar = 100 μm.

Supplementary Table 1. Antibodies and fluorescent dyes for organoid labelling

Antibody	Species	Dilution	Manufacturer	Cat. no.
DAPI	-	1:1000	Invitrogen	D1306
Cytokeratin 7	rabbit (rb)	1:300	Abcam	Ab181598
E-cadherin	Rat	1:150	Invitrogen	14-3249-82
HLA-G	mouse (ms)	1:150	Abcam	ab52454
β-hCG	Rabbit	1:300	Abcam	ab243581
SDC-1	Rabbit	1:500	Abcam	ab128936
Anti-Rb Alexa Fluor 488	Goat	1:250	Abcam	ab150077
Anti-Ms Alexa Fluor 594	Goat	1:250	Abcam	ab150120
Anti-Rat Alexa Fluor 647	Goat	1:250	Abcam	ab150167

Supplementary Table 2. Proteomics reagents

Reagent Name	Chemical components
SDC Master Mix	100mM HEPES pH 8.5, 1% sodium deoxycholate (SDC), 200mM tributylphosphine (TBP) in water, 1000mM acrylamide in water, 1000mM Dithiothreitol (DTT)
Trypsin solution	1ug/ul Trypsin Gold in 50mM Acetic acid
Load Buffer	90% Acetonitrile, 1.0% Trifluoroacetic Acid
Wash Buffer	10% Acetonitrile, 0.1% Trifluoroacetic Acid
Elution Buffer	71uL 1M NH_4OH, 800uL of 100% Acetonitrile, 129uL water
MS Loading Solvent	2% acetonitrile, 0.2% trifluoroacetic acid
