# Supplementary material for: Matrix directs trophoblast differentiation in a bioprinted organoid model of early placental development
Source: Nat Commun. 2025 Sep 12;16:8267. doi: 10.1038/s41467-025-62996-0 (PMC12432263; doi:10.1038/s41467-025-62996-0)
Supplement: Supplementary file 2 — Description of Additional Supplementary Files [file 41467_2025_62996_MOESM2_ESM.pdf]

Title: Supplementary Movie 1

Description: ACH-3P cell organoid formation in Matrigel by time lapse microscopy.

Title: Supplementary Movie 2

Description: ACH-3P cell organoid formation in bioprinted matrix by time lapse microscopy.

Title: Supplementary Movie 3

Description: 3D rendering of ACH-3P bioprinted organoid reveals internal syncytialisation.
