# Supplementary material for: Matrix directs trophoblast differentiation in a bioprinted organoid model of early placental development
Source: Nat Commun. 2025 Sep 12;16:8267. doi: 10.1038/s41467-025-62996-0 (PMC12432263; doi:10.1038/s41467-025-62996-0)
Supplement: Supplementary file 6 — Reporting Summary [file 41467_2025_62996_MOESM6_ESM.pdf]

nature portfolio

Corresponding author(s): Professor Lana McClements

Last updated by author(s): Oct 3, 2024

Reporting Summary

Nature Portfolio wishes to improve the reproducibility of the work that we publish. This form provides structure for consistency and transparency in reporting. For further information on Nature Portfolio policies, see our Editorial Policies and the Editorial Policy Checklist.

Statistics

For all statistical analyses, confirm that the following items are present in the figure legend, table legend, main text, or Methods section.

n/a	Confirmed	
☐	☒	The exact sample size (n) for each experimental group/condition, given as a discrete number and unit of measurement
☐	☒	A statement on whether measurements were taken from distinct samples or whether the same sample was measured repeatedly
☐	☒	The statistical test(s) used AND whether they are one- or two-sided<br>*Only common tests should be described solely by name; describe more complex techniques in the Methods section.*
☒	☐	A description of all covariates tested
☐	☒	A description of any assumptions or corrections, such as tests of normality and adjustment for multiple comparisons
☐	☒	A full description of the statistical parameters including central tendency (e.g. means) or other basic estimates (e.g. regression coefficient) AND variation (e.g. standard deviation) or associated estimates of uncertainty (e.g. confidence intervals)
☐	☒	For null hypothesis testing, the test statistic (e.g. F, t, r) with confidence intervals, effect sizes, degrees of freedom and P value noted<br>*Give P values as exact values whenever suitable.*
☒	☐	For Bayesian analysis, information on the choice of priors and Markov chain Monte Carlo settings
☒	☐	For hierarchical and complex designs, identification of the appropriate level for tests and full reporting of outcomes
☒	☐	Estimates of effect sizes (e.g. Cohen's d, Pearson's r), indicating how they were calculated

Our web collection on statistics for biologists contains articles on many of the points above.

Software and code

Policy information about availability of computer code

Data collection	IncuCyte (v 2022B.rev2), NIS Elements Advanced Research software (v. 5.30.06), Illumina Conversion Software (bcl2fastq v2.19.0.316), 10X Genomics cellranger (v3.1.0), Peaks Studio (v11)
Data analysis	ImageJ (v1.53j), Bitplane Imaris (v9.6.0), R (v4.3.2), Monocle 3 R package (v1.3.4), Microsoft Excel (v2016), GraphPad Prism (v9.4.1), Seurat (v5.1.0)

For manuscripts utilizing custom algorithms or software that are central to the research but not yet described in published literature, software must be made available to editors and reviewers. We strongly encourage code deposition in a community repository (e.g. GitHub). See the Nature Portfolio guidelines for submitting code & software for further information.

Data

Policy information about availability of data

All manuscripts must include a data availability statement. This statement should provide the following information, where applicable:

- Accession codes, unique identifiers, or web links for publicly available datasets
- A description of any restrictions on data availability
- For clinical datasets or third party data, please ensure that the statement adheres to our policy

All the data generated or analysed during this study are available from the corresponding author upon reasonable request. (typographical error detected that will be edited in revision)

Research involving human participants, their data, or biological material

Policy information about studies with human participants or human data. See also policy information about sex, gender (identity/presentation), and sexual orientation and race, ethnicity and racism.

Reporting on sex and gender	The sex of the cell line was included to account for any sex-specific effects.
Reporting on race, ethnicity, or other socially relevant groupings	We did not have access to or include any racial information regarding the cell line used.
Population characteristics	N/A
Recruitment	N/A
Ethics oversight	University of Technology Sydney

Note that full information on the approval of the study protocol must also be provided in the manuscript.

Field-specific reporting

Please select the one below that is the best fit for your research. If you are not sure, read the appropriate sections before making your selection.

[x] Life sciences [ ] Behavioural & social sciences [ ] Ecological, evolutionary & environmental sciences

For a reference copy of the document with all sections, see nature.com/documents/nr-reporting-summary-flat.pdf

Life sciences study design

All studies must disclose on these points even when the disclosure is negative.

Sample size	Various sample sizes of organoid numbers were used for different experiments based on our previous experience with 3D in vitro models.
Data exclusions	No data were excluded in analyses.
Replication	Organoid generation from Matrigel or bioprinted conditions was assessed following multiple experimental repeats using different passages. This extended to downstream analyses.
Randomization	Cells used in both Matrigel-embedding and bioprinting were always taken from the same passage of cells when comparing conditions. Sample replicates were randomly processed by LC-MS/MS for proteomic profiling to mitigate for any measurement bias.
Blinding	No blinding was relevant for this study.

Reporting for specific materials, systems and methods

We require information from authors about some types of materials, experimental systems and methods used in many studies. Here, indicate whether each material, system or method listed is relevant to your study. If you are not sure if a list item applies to your research, read the appropriate section before selecting a response.

Materials & experimental systems

n/a	Involved in the study	
[ ]	[x]	Antibodies
[ ]	[x]	Eukaryotic cell lines
[x]	[ ]	Palaeontology and archaeology
[x]	[ ]	Animals and other organisms
[x]	[ ]	Clinical data
[x]	[ ]	Dual use research of concern
[x]	[ ]	Plants

Methods

n/a	Involved in the study	
[x]	[ ]	ChIP-seq
[x]	[ ]	Flow cytometry
[x]	[ ]	MRI-based neuroimaging

Antibodies

Antibodies used	Cytokeratin 7 (Abcam, ab181598, Lot:GR3321316-3)<br>E-cadherin (Invitrogen, 14-3249-82, Clone: DECMA-1, Lot:2340002)<br>HLA-G (Abcam, ab52454, Lot: GR3372627-5)<br>B-hCG (Abcam, ab243581, Lot: GR3402861-4)<br>SDC-1 (Abcam, ab128936, 1014070-10)

Anti-Rabbit Alexa Fluor 488 (Abcam, ab150077, GR3376391-5)
Anti-Mouse Alexa Fluor 594 (Abcam, ab150120, Lot: GR3378537-5)
Anti-Rat Alexa Fluor 647 (Abcam, ab150167, Lot: GR3384463-5)

Validation	These are commercially available antibodies from reputable sources that have a number of publications associated with their research use.

Eukaryotic cell lines

Policy information about cell lines and Sex and Gender in Research

Cell line source(s)	The ACH-3P cell line was custom made by Professor Gernot Desoye (Graz Medical University, Austria). The cell line is male and was characterised in a publication that was referenced within our manuscript.
Authentication	The cell line was characterised and authenticated by its creators in referrenced publications provided within the manuscript.
Mycoplasma contamination	All cells used in the study were confirmed negative for mycoplasma prior to use.
Commonly misidentified lines (See ICLAC register)	n/a

Plants

Seed stocks	n/a
Novel plant genotypes	n/a
Authentication	n/a
